# Supplementary material for: Impact of Amiodarone Therapy on the Ablation Outcome of Ventricular Tachycardia in Arrhythmogenic Right Ventricular Cardiomyopathy
Source: J Clin Med. 2022 Dec 7;11(24):7265. doi: 10.3390/jcm11247265 (PMC9787968; doi:10.3390/jcm11247265)
Supplement: Supplementary file 1 [file jcm-11-07265-s001.zip › jcm-2056331-supplementary.pdf]

Supplemental Table S1. Comparison of substrate characteristics between patients with different periods on amiodarone before ablation

	Amiodarone > 6 months (N = 21)	Amiodarone more than 8 weeks and ≤ 6 months (N = 22)	*p*-value
**RV endocardium**			
Bipolar voltage*	1.8 ± 0.6	2.0 ± 0.7	0.222
Unipolar voltage*	4.9 ±1.7	4.9 ± 1.2	0.999
Total activation time (ms)	167.4 ± 27.2	161.3 ±34.2	0.496
Bipolar low voltage zone (cm^2)	40.4 ±32.3	29.2± 20.9	0.185
Bipolar low voltage zone, %	17.3± 12.9	13.6± 8.0	0.262
Bipolar scar (cm^2)	19.5 ± 15.8	15.4 ± 11.2	0.332
Bipolar scar, %	8.8 ± 6.6	7.3 ± 4.3	0.385
Unipolar low voltage zone (cm^2)	65.2 ± 34.2	65.4 ± 21.2	0.977
Unipolar low voltage zone, %	26.6 ± 12.8	26.6 ±7.9	0.999
Area with abnormal electrograms	17.7 ± 17.4	15.8 ± 13.7	0.692
**RV epicardium**	**N=16**	**N=19**	
Bipolar voltage*	1.5 ± 1.0	1.1 ± 0.4	0.152
Total activation time (ms)	212.3 ± 31.6	210.3 ±21.0	0.825
Bipolar low voltage zone (cm^2)	97.6± 53.9	123.2 ±56.0	0.180
Bipolar low voltage zone, %	33.1± 22.3	38.9 ±18.8	0.410
Bipolar scar (cm^2)	51.1 ± 34.0	61.2 ± 34.6	0.390
Bipolar scar, %	17.4 ± 13.4	18.6 ± 9.7	0.756

Area with abnormal electrograms	24.7 ± 30.1	31.8 ± 28.1	0.434

*The average of bipolar or unipolar median voltage.

ARVC, arrhythmogenic right ventricular cardiomyopathy; RV, right ventricular; VT, ventricular tachycardia

“
